# Uric acid as a prognostic predictor in COVID-19

**DOI:** 10.12669/pjms.38.8.6636

**Published:** 2022

**Authors:** Ergün Parmaksız, Elif Torun Parmaksız

**Affiliations:** 1 Ergün Parmaksız, Department of Nephrology, Kartal Dr Lutfi Kırdar City Hospital, Health Sciences University, Istanbul, Turkey.; 2 Elif Torun Parmaksız Department of Chest Diseases, Sancaktepe İlhan Varank Training Hospital, Health Sciences University, Istanbul, Turkey

**Keywords:** Uric acid, COVID-19, Prognosis

## Abstract

**Objective:**

The purpose of our study was to investigate the incidence and prognostic significance of baseline and control uric acid values in COVID-19.

**Methods:**

The study population included patients admitted with the diagnosis of SARS-CoV-2 between March 2020 and March 2021. The demographic data, clinical, laboratory, and radiological findings were recorded. Uric acid levels were measured at the time of admission for 498 patients and at the most severe period of the disease in 143 patients. Length of hospital stay, need for admission to intensive care unit, the course, and outcomes during hospitalization were recorded.

**Results:**

The mean age of 261 male and 207 female patients was 62.7(21-95) years. At the time of admission, 21 patients had hypouricemia and 170 had hyperuricemia. The need for ICU was 47.6% in the hypouricemic, 19.2% in the normouricemic, and 21.2% in the hyperuricemic groups. The mean uric acid level was 5.24±2.54 mg/dl in patients who required ICU admission and 5.18±1.98 mg/dl in patients who were discharged from the ward. The difference was not statistically significant. The mean uric acid level was not significantly different in the deceased and survivors. In 143 subjects, uric acid levels were measured after the progression of COVID-19; 73 of them were admitted to the ICU. The mean uric acid levels were found to be significantly decreased in patients with a negative prognosis

**Conclusion:**

In our study, hypouricemia was not found to be a major feature of SARS-CoV-2 infection. Low baseline uric acid levels were associated with increased ICU admission. The decline in uric acid levels during hospital stay predicted poor prognosis, as well.

## INTRODUCTION

Severe Acute Respiratory Syndrome Coronavirus 2 (SARS-CoV-2) first emerged in December 2019 as a public health crisis in Wuhan city, Hubei province, China, and then became a global problem. Coronavirus disease 2019 (COVID-19) can occur in a wide clinical range, from mild symptoms such as fever, cough or fatigue to severe pneumonia, septic shock, organ failure or death.^1^

Uric acid is an end-product of purine metabolism. It is mainly synthesized by the liver, intestines and the vascular endothelium; dissolved in the blood, and excreted from the body through urine.^2^ Low uric acid levels may be secondary to disorders of liver or kidney metabolism. Studies have shown that COVID-19-related kidney damage may be characterized by increased levels of proteinuria, hematuria, and serum creatinine.^3^,^4^ It has been suggested that particularly proximal tubules are affected. Proximal tubule damage increases uric acid excretion, leading to a decrease in serum uric acid levels.^5^ Given the importance of serum uric acid levels in terms of showing the extent of kidney damage, we believe that they may be associated with the clinical presentation, course and consequences of COVID-19 infection. We suspected that uric acid levels might predict COVID-19 prognosis, and the role of uric acid values in COVID-19 needs to be investigated. In our study, we aimed to evaluate the effect of uric acid levels on clinical presentation, course and outcomes of COVID-19.

## METHODS

**Fig.1:**
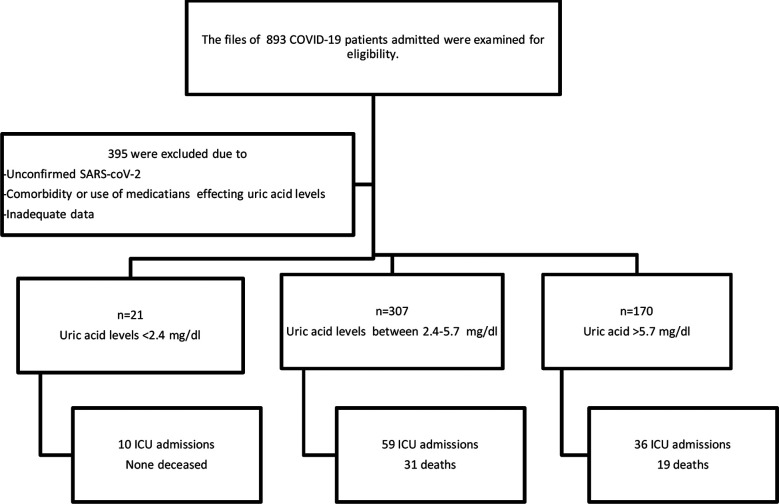
The flowchart of patient selection and classification.

We conducted this retrospective observational study on patients admitted to our hospital with diagnosis of SARS-CoV-2 between March 2020 and March 2021. The electronic medical records were reviewed and COVID-19 patients who underwent uric acid analysis at the time of admission were included (Fig.1). The demographic data, clinical, laboratory and radiological findings were recorded. In subjects who were admitted to the ICU, uric acid levels at the time of hospital admission and at the time of ICU admission were recorded. If the subjects were discharged without ICU admission, the uric acid values at the time of hospital admission and during the most severe period of the disease were recorded. The most severe period of COVID-19 was considered when maximum oxygen support was required and predictors associated with poor prognosis, namely CRP, procalcitonin, ferritin and D-dimer were at highest values. Uric acid levels between 2.5-5.6 mg/dl were considered normal. The study was approved by the Turkish Republic Ministry of Health and local ethics committee.

Diagnosis of COVID-19 infection was confirmed by a positive SARS-CoV-2 reverse transcriptase polymerase chain reaction (RT-PCR) test or the presence of ICD-10 code U07.1 based on clinical, laboratory and radiological findings. Throat-swab specimens were obtained from the oropharynx and nasopharynx from all patients at admission and were put in viral-transport medium for RT-PCR test.

Hospital admission was defined as patient presence and treatment in a dedicated COVID-19 unit in hospital. Fasting blood samples were collected from all patients in the first morning following admission and sent for laboratory tests.

The data from a total of 893 patients admitted between March 2020 and March 2021 with COVID-19 were initially screened. The subjects with incomplete medical records, cases with known diagnosis of gout, hyperparathyroidism, renal failure or nephropathy, renal transplantation, decompensated heart failure and cases prescribed with drugs that will affect uric acid levels such as diuretics, antineoplastic or antituberculosis drugs, theophylline, levodopa were not included in the study population (Fig.1).

### Data collection:

The demographic data were collected. Data regarding the clinical symptoms on admission (i.e., cough, fever, dyspnea, fatigue, headache, chest pain, gastrointestinal symptoms such as nausea, vomiting, or diarrhea) and comorbidities (i.e., hypertension, cardiovascular disease, chronic respiratory disease) were extracted from medical records. The physical examination findings on initial admission were recorded; these included fever, blood pressure, heart rate, and oxygen saturation. The laboratory tests included complete blood count, fasting blood glucose, C-reactive protein (CRP), procalcitonin, D-dimer, ferritin, lactate dehydrogenase, renal and hepatic function tests, electrolytes, and uric acid. CT scan examinations were done for all inpatients. The radiological findings were classified as no involvement, unilateral involvement, or bilateral involvement. Length of hospital stay, need for admission to intensive care unit, the course, and outcomes during hospitalization were recorded. Treatment protocols were applied according to the COVID-19 management guideline of the Turkish Ministry of Health.^6^

### Statistical Analysis:

Categorical variables were described as numbers (%). Continuous variables were analyzed parametrically by means and standard deviations, median, minimum and maximum. Differences between categorical variables were calculated using the chi-square method. Differences between continuous variables were calculated using the T-test and ANOVA test. Pearson analysis was used for correlation tests. *A P-*value less than 0.05 was considered statistically significant. Statistical analyses were performed using SPSS software (version 17.0).

## RESULTS

Data were collected from 498 patients; 261(52.4%) were male. The mean age of the study population was 62.7(21-95) years. The demographic characteristics and comorbidities are shown in Table-I.

**Table-I:** The general characteristics of the patients.

Number of cases	498
Gender (Female/Male)	237/261
***Age (years)***	
Min-Max(Median)	21-95 (65)
Mean±SD	62.78±15.63
***Comorbidities***	***n (%)***
DM	277 (56)
Hypertension	259 (52)
Cardiovascular disease	129 (26)
Chronic lung disease	59 (12)

SD: Standard deviation, DM: Diabetes mellitus.

The most common complaints on admission were shortness of breath (n=254, 52%), fever (n=233, 48%), cough (n=224, 46%), fatigue (n=184, 38%), and chest pain (n=72, 15%). At the time of admission, 21 patients had hypouricemia and 170 had hyperuricemia. The distribution of symptoms, physical findings, and comorbidities is demonstrated in Table-II.

**Table-II:** The demographic characteristics of the study population

	Hypouricemic group	Normouricemic group	Hyperuricemic group	p
Number of cases	21	307	170	
***Age (years)***				
Min-Max(Median)	27-82 (56)	20-91 (60)	19-95 (71)	<0.001
Mean±SD	56.76±14.00	59.92±15.63	68.70±14.09	
Gender (female/male)	9/12	154/153	74/96	0.34
***Comorbidities***	***n (%)***	***n (%)***	***n (%)***	
Hypertension	3 (14.3)	141 (45.9)	115 (67.6)	<0.001
DM	12(57.1)	164 (53.4)	101 (59.4)	0.44
Chronic lung disease	3 (14.3)	28 (9.1)	28 (16.5)	0.05
Cardiovascular disease	3 (14.3)	64 (20.8)	62 (36.5)	<0.001
***Presenting symptoms***	***n (%)***	***n (%)***	***n (%)***	
Cough	10(47.6)	152(49.5)	62(36.5)	0.022
Fever	12(57.1)	155(50.5)	66(38.8)	0.022
Chest thightness	13(61.9)	143(46.6)	98(57.6)	0.019
Fatigue	7(33.3)	121(39.4)	56(32.9)	0.40
Nausea, diarrhea	3(14.3)	50(16.3)	37 (21.8)	0.31
***Body temperature (ºC)***				
Min-Max(Median)	36.0-39.0(37.5)	36.0-39.0(36.8)	35.2-39.7(36.7)	0.002
Mean±SD	37.4±1.00	37.0±0.71	36.8±0.61	
***Systolic blood pressure (mmHg)***				
Min-Max(Median)	90-130(110)	80-220(120)	80-180(120)	
Mean±SD	110.00±12.47	117.65±16.01	117.67±14.66	0.107
***Diastolic blood pressure (mmHg)***				
Min-Max(Median)	60-85(70)	40-110(70)	50-110(70)	0.547
Mean±SD	70.00±8.66	72.22±8.77	72.30±8.73	
***Heart rate (beat/minute)***				
Min-Max(Median)	72-113(94)	50-133(83)	52-140(80)	0.034
Mean±SD	93.05±11.98	86.54±15.17	84.23±14.93	
***Arterial oxygen saturation (%)***				
Min-Max(Median)	75-99(93)	64-99(94)	60-99(94)	
Mean±SD	90.95±6.94	92.44±5.18	91.46±6.59	0.153

SD: Standard deviation, DM: Diabetes mellitus.

The laboratory findings of the study population are summarized in Tables III and IV. In the hypouricemic group, 10(47.6%) required ICU admission. The need for ICU was 19.2% in the normouricemic and 21.2% in the hyperuricemic groups.

**Table-III:** The laboratory findings of the study population

	Min-Max (Median)	Mean±SD
Uric acid (mg/dl)	1.60-16.60(4.80)	5.20±2.11
Creatinine(mg/dl)	0.26-6.07(0.87)	1.28±1.36
Urea(mg/dl)	10-282(37)	50.68±40.33
Leucocytes(/uL)	1800-69200(7000)	8122±5151
Lymphoctes(/mm³)	100-6600(1100)	1296±776
Hemoglobin(g/dL)	5.60-17.00(12.30)	12.09±2.17
Platelets(/uL)	15000-859000(210000)	226813±98637
ALT(U/L)	1.-269(22)	31.33±34.21
AST(U/L)	3-598(28)	38.38±48.03
LDH(U/L)	32-1473(264)	297.43±147.71
Sodium(mmol/L)	114-153(136)	135.30±4.73
Potassium(mmol/L)	2.46-6.69(4.20)	4.19±0.57
Albumin(g/L)	18-50(35.00)	34.02±5.77
CRP(mg/L)	2.98-460.00(40.00)	65.84±72.48
Procalcitonin(µg/L)	0.02-73.22(0.12)	0.94±4.64
D-dimer(µg/L)	170-30000(1000)	2003±3111
Ferritin(µg/L)	6.20-10000.00(235.65)	435.10±670.46

ALT, Alanine transferase; AST, Aspartate transferase; LDH, Lactate dehydrogenase; CRP, C-reactive protein.

**Table-IV:** The laboratory findings of the groups based on uric acid levels

	Hypouricemic group	Normouricemic group	Hyperuricemic group	p
***WBC(/uL)***				
Min-Max(Median)	1800-19000(6100)	1900-25000(7700)	2500-71200(8400)	<0.001
Mean±SD	7338±4558	7483±3765	9374±6902	
***Lymphoctes(/mm³)***				
Min-Max(Median)	100-5400(1400)	100-4300(1100)	300-6600(1100)	0.314
Mean±SD	1480±1413	1315±718	1238±770	
***Hemoglobin(g/dL)***				
Min-Max(Median)	7.30-14.70(12.90)	6.40-17.00(11.50)	5.60-16.90(11.50)	<0.001
Mean±SD	12.29±1.85	12.44±2.06	11.46±2.27	
***Platelets(/uL)***				
Min-Max(Median)	17000-859000(210000)	22000-	13000-	0.356
Mean±SD		613000(210000)	748000(212000)	
	256333±172111	226606±94610	223500±93591	
***LDH(U/L)***				
Min-Max(Median)	190-466(258)	32-769(255)	120-1473(268)	0.463
Mean±SD	306.89±99.70	290.60±109.29	308.83±203.98	
***Sodium(mmol/L)***				
Min-Max(Median)	128-143(136)	114-149(136)	122-153(135)	0.891
Mean±SD	135.75±4.78	135.24±4.74	135.34±7.46	
***Potassium(mmol/L)***				
Min-Max(Median)	3.10-5.20(4.10)	2.46-5.90(4.47)	2.77-6.69(4.30)	<0.001
Mean±SD	3.88±0.49	4.14±0.49	4.32±0.68	
***CRP(mg/L)***				
Min-Max(Median)	3.00-198.00(21.90)	2.98-480.00(45.90)	3.00-281.00(61.70)	0.702
Mean±SD	77.93±66.29	64.51±78.00	66.71±62.54	
***Procalcitonin(µg/L)***				
Min-Max(Median)	0.02-2.09(0.07)	0.02-33.15(0.16)	0.02-73.22(0.28)	0.244
Mean±SD	0.32±0.60	0.66±2.69	1.49±6.95	
***D-dimer(µg/L)***				
Min-Max(Median)	290-176400(575)	170-30000(1340)	170-30000(1195)	0.306
Mean±SD	2448±4008	1824.38±2959.06	2250.60±3237.34	
***Ferritin(µg/L)***				
Min-Max (Median)	9.60-2000(162.00)	6.20-10000.00(205.75)	11.90-2638.00(262.20)	0.574
Mean±SD	482.71±561.86	460.61±786.58	338.45±448.52	

*One way ANOVA test.

The mean uric acid level was 5.24±2.54 mg/dl in patients who required ICU admission and 5.18±1.98 mg/dl in patients who were discharged from the ward. The difference was not statistically significant (p=0.79). The mean uric acid level was not significantly different in the deceased and survivors (5.75±2.84 mg/dl and 5.14±2.01 mg/dl, respectively) (p=0.14).

In 143 subjects, uric acid levels were measured after the progression of COVID-19; 73 of them were admitted to the ICU, and uric acid measurements were done at the time of ICU admission. In 70 patients, uric acid levels during the most severe period of the disease were recorded. The most severe period of COVID-19 was considered when maximum oxygen support was required and predictors associated with poor prognosis, namely CRP, procalcitonin, ferritin, and D-dimer were at the highest values. The mean uric acid levels were found to be significantly decreased in patients with a negative prognosis (Table-V).

**Table-V:** The change in laboratory measurements with progression of the disease severity.

	Mean±SD		P value

	On admission	During most severe period	
Uric acid (mg/dl)	5.51±2.38	3.90±2.16	<0.001
Creatinine (mg/dl)	1.37±1.35	2.08±11.23	0.21
Urea (mg/dl)	59.90±48.99	54.52±46.99	0.47
Sodium (mmol/L)	134.70±5.23	137.24±6.95	<0.001
Potassium (mmol/L)	4.17±0.64	3.99±0.63	0.014

Correlation analyses revealed a negative correlation between uric acid levels and ferritin (r=-0.03; p=0.53) and length of hospital stay (r=-0.03; p=0.41), but the correlation was not statistically significant. On the other hand, uric acid levels measured when the disease got more severe were significantly correlated with length of hospital stay (r=-0.20; p=0.01).

## DISCUSSION

In this analysis, we studied uric acid levels in hospitalized COVID-19 patients. Intriguingly, hypouricemia was not found to be a major feature of SARS-CoV-2 infection. However, our findings provide novel evidence on the role of uric acid in the progress and outcomes of the infection. We have observed that low baseline uric acid levels were associated with increased ICU admission. Uric acid levels at hospital admission were found to predict the probability of ICU requirement in COVID-19. We have also showed that a decline in uric acid levels during hospital stay predicted a poor prognosis. The significance of the change in uric acid levels during the course of the disease is an important novel finding.

Uric acid plays a well-established causative role in inflammatory arthritis, gout, and metabolic syndrome; on the other hand, it has several paramount physiological functions. It is a prominent anti-oxidant and constitutes the majority of the antioxidant capacity of blood plasma, as well as the respiratory tract. It is involved in improving endothelial functions and immune response. It plays a protective role against infections and autoimmune diseases.^7^ In the kidney, uric acid is filtered from glomeruli in the kidneys, and eventually, 90% is reabsorbed in the proximal tubules into the blood capillaries. In a recent report, Werion et al., have clearly documented proximal renal tubule dysfunction in COVID-19. Electron microscopic analyses revealed prominent proximal tubular damage, including loss of brush border, acute tubular necrosis, intraluminal debris, and a marked alteration in the megalin expression.^5^ Hypouricemia was due to defective reabsorption leading to inappropriate uricosuria. The authors have compared the clinical pictures in this patient population and have come across a significant independent link between hyperuricosuria and increased risk of invasive mechanical ventilation or mortality.^5^ They have also strengthened the findings by showing prominent and diffuse proximal tubular damage in postmortem examination of six deceased cases.

In a study published in 2005, much before the emergence of SARS-CoV-2, uricosuria resulting in hypouricemia was found to be present in about one-fourth of SARS-CoV patients and this subset had a poor prognosis, compared to normouricemic counterparts.^8^ Similar evidence was derived from a study concerning SARS-coV-2; extremely low serum uric acid levels have been found to be associated with poor prognosis in COVID-19. The patients with severe infection tended to have significantly lower uric acid levels than mild cases.^9^

### Limitation of the study:

It is the lack of data about the uric acid levels of these patients prior to the infection. For instance, cardiovascular diseases and hypertension might cause endothelial dysfunction, thereby causing elevated baseline uric acid levels. Still, we suppose we have overcome this handicap by measuring the decline in serum uric acid values during the course of the infection. Another limitation is that we did not measure urinary uric acid levels, therefore we cannot comment on uricosuria in the study population.

All our patients received treatment protocols in accordance with the COVID-19 management guideline of the Turkish Ministry of Health.^6^ The treatment regimens included favipiravir, an antiviral agent with a wide spectrum. This drug is a purine nucleic acid analog and can cause serum uric acid elevation by decreasing urinary excretion. This effect is almost always reversible and subclinical.^10^ We do not suppose this treatment to have an impact on the study results because a similar treatment is applied for all the study population.

Our findings suggest that a decline in serum uric acid in COVID-19 may predispose to more severe disease. Hypouricemia is associated with an enhanced risk of severe infection. It is important to emphasize that low baseline uric acid levels, as well as a decline in uric acid levels during the hospitalization period, constitute a higher risk for poor prognosis.

### Authors’ Contribution:

**EP** conceived, designed and did data collection, and final approval of manuscript.

**ETP** did statistical analysis & editing of manuscript, and manuscript writing.
